# Harmonized Integration of GWO and J-SLnO for Optimized Asset Management and Predictive Maintenance in Industry 4.0

**DOI:** 10.3390/s25092896

**Published:** 2025-05-03

**Authors:** A. N. Arularasan, P. Ganeshkumar, Mohammad Alkhatib, Tahani Albalawi

**Affiliations:** 1 Department of Computer Science and Engineering, B.S. Abdur Rahman Crescent Institute of Science and Technology, Vandalur, Chennai 600 048, Tamil Nadu, India; arularasan@live.com; 2 Department of Computer Science, College of Computer and Information Sciences, Imam Mohammad Ibn Saud Islamic University (IMSIU), Riyadh 11432, Saudi Arabia; gpperumal@imamu.edu.sa (P.G.); mhalkhatib@imamu.edu.sa (M.A.)

**Keywords:** Industry 4.0, optimization algorithms, predictive maintenance, Grey Wolf Optimization (GWO), J-SLnO

## Abstract

**Highlights:**

**What are the main findings?**

- The study shows that, in terms of execution speed, cost-effectiveness, and energy usage, GWO and J-SLnO perform better than conventional scheduling algorithms.
- Compared to GWO, J-SLnO performs predictive maintenance activities with greater accuracy and stability.

**What is the implication of the main finding?**

- In Industry 4.0, the suggested optimization strategies improve the effectiveness of predictive maintenance and asset management.
- J-SLnO is a dependable option for practical industrial applications that demand endurance and excellent prediction accuracy.

**Abstract:**

The study encompasses the application of two different advanced optimization algorithms on asset management and predictive maintenance in Industry 4.0—Grey Wolf Optimization and Jaya-based Sea Lion Optimization (J-SLnO). Using this derivative, the authors showed how these techniques could be combined through resource scheduling techniques to demonstrate drastic improvement in the level of efficiency, cost-effectiveness, and energy consumption, as opposed to the standard MinMin, MaxMin, FCFS, and Round Robin. In this sense, GWO results in an execution time reduction between 13 and 31%, whereas, in J-SLnO, there is an execution time reduction of 16–33%. In terms of cost, GWO shows an advantage of 8.57–9.17% over MaxMin and Round Robin, based on costs, while J-SLnO delivers a better economy for the range of savings achieved, which is between 13.56 and 19.71%. Both algorithms demonstrated tremendous energy efficiency, according to the analysis, which showed 94.1–94.2% less consumption of energy than traditional methods. Moreover, J-SLnO was reported to be more accurate and stable in predictability, making it an excellent choice for accurate and more time-trusted applications. J-SLnO is being increasingly recognized as a powerful yet realistic solution for the application of Industry 4.0 because of efficacy and reliability in predictive modeling. Not only does this research validate these optimization techniques to better use in practical life, but it also extends recommendations for putting the techniques into practice in industrial settings, thus laying the foundation for smarter, more efficient asset management and maintenance processes.

## 1. Introduction

The industrial sector is rapidly evolving through revolutionary next-gen computing technologies—cloud-edge computing, big data, the Internet of Things (IoT), and cyber-physical systems (CPSs). Smart manufacturing—the promise of any future industrial operation—stands on the permanent transmission and continuous analysis of strategizing data within shop floors with the goal of maximizing efficiency and productivity. One of the great enablers of the transformation is the Industrial Internet of Things (IIoT), which is the extension of IoT applications to industrial environments, enabling a real-time collection of data and control of manufacturing processes [1]. An immense volume of data are generated from such environments every second. Sample collection for healthcare product manufacturers is estimated to encompass 5000 sample collections every 33 ms, thus summing it up to over 4 trillion samples in a year [2]. In the same manner, the industrial facility with ten cameras and a hundred machine tools is likely to generate data approximately to 72 terabytes every year [3].

However, this increasing volume faced many challenges for traditional in-house servers, which lack the storage, memory, and processing power to cope with such great information. Because of this, many industries have leaned toward cloud computing, which has high scalability in resources, as well as advanced data analytics. But experiments conducted revealed that the increasing distance from IIoT devices to cloud data centers leads to network latency and overhead bandwidth consumption, thereby impeding real-time applications [4]. Fog computing is offering relief from this ordeal by bringing the processing closer to the source instead of everything being sent to the cloud. Therefore, fog computing possesses all the advantages of both edge and cloud computing; fog computing minimizes latency, lessens network congestion, and improves industrial time-sensitive application performance.

These changes in manufacturing have brought about an increase in the use of tracking technologies, with RFID, BLE tags, 1D barcodes, QR codes, and LoRa tags being the adopted technologies due to their cost-effectiveness and relative ease of integration. The concept of the reference architecture model for the Internet of Things (IoT), or smart manufacturing systems, is basically defined by RAMI 4.0, and it has found its application in the area of industrial automation under the OPC UA standard communication protocol. It has enabled efficient data exchange among industrial assets and hence communication of different manufacturing system components.

### 1.1. Challenges in Industrial Maintenance

Maintenance is now given serious consideration in industrial sectors since Industry 4.0 came into being, particularly because operating costs and efficiency depend on extending capital equipment life [5,6]. PdM is fundamental to preventing industrial equipment failures in manufacturing, automotive, and aviation industries [7,8,9]. With the utilization of data analytics and health factor assessment, the early detection of potential failures becomes possible with PdM [10,11,12]. Hence, PdM reduces accidents and financial losses. Conventional maintenance strategies have their limitations; preventive maintenance and reactive maintenance both have their own limitations. In preventive maintenance service, maintenance is undertaken based on set time periods without any reference to the actual condition of the equipment; such unnecessary servicing can sometimes result in missing critical failures [13]. Reactive maintenance, on the contrary, only acknowledges failures past their occurrence, which can prove costly in terms of downtime and operational interruption.

On the other hand, PdM allows condition monitoring and fault prediction well before they become critical. In this way, the operational life can be prolonged through timely component replacements and repairs [14,15]. Furthermore, facilities use sensors for monitoring, perform periodic inspections to validate the effectiveness of predictive analysis and gather vital data [16,17]. Further understanding is achieved using digital tools such as Computer-Aided Facility Management (CAFM) and Computerized Maintenance Management Systems (CMMS), which enhance maintenance processes toward greater efficiency [18,19,20,21,22]. However, data are frequently manually transferred between maintenance systems, often resulting in delays and inefficiencies that diminish the full impact of PdM [23].

### 1.2. Gaps and Shortcomings of Existing Approaches

While the impact of modern technologies like building information modeling (BIM) has improved maintenance records and facility management, massive gaps still exist for integrating data-driven decision-making [24,25,26]. IoT-enabled sensor networks allow real-time collection of data, but the challenge of seamlessly integrating data from multiple sources is still not overcome. In the past, prototypes of decision support systems for maintenance were introduced, but many go their own way regarding data processing and analytics, creating fragmented maintenance strategies not unifying with any of their solutions [27].

Researchers suggest that machine learning will have a great influence in the foreseeable future [28,29]; hence, its use in predictive maintenance is quite promising. With the ability to process historical and real-time data to make accurate predictions about potential equipment failures, machine-learning algorithms offer decision support. These advances notwithstanding, predictive modeling still requires additional development work, including the refinement of integrations of the data, enhancement of maintenance methodologies, and maximum ease of application within smart manufacturing environments.

### 1.3. Aim and Scope

Industry 4.0 significantly boosts manufacturing technology efficiency by leveraging real-time data gathering and examination. The whole connectivity facilitated via IIoT allows businesses to track assets promptly and accurately using IIoT sources and associated information services. The vast amount of industrial data, measured through IIoT sensors, greatly enhances data visibility. A main focus within manufacturing is the predictive analysis of equipment conditions, necessitating a robust and efficient approach to managing the vast data streams. In this context, the GWO and J-SLnO algorithms emerge as promising methods due to their notable advantages.

The key objectives of this research paper are as follows:

- Introduces a resource scheduling approach involving GWO and J-SLnO algorithms for effective asset management within the framework of Industry 4.0.
- Conducted simulations by means of a variety of scheduling techniques, including MinMin, MaxMin, FCFS, RoundRobin, and the Grey Wolf Optimization (GWO) and Jaya-based Sea Lion Optimization (J-SLnO) algorithms. Crucial factors such as execution time, cost-effectiveness, and energy usage were taken into consideration when conducting the evaluation.
- Enhanced the manufacturing process‘s decision support system (DSS) through the incorporation of optimized procedures, with a focus on predictive maintenance tactics.
- The research delves into how these optimization strategies improve the predicted accuracy and efficiency of recognizing possible equipment problems. By optimizing model parameters, GWO or J-SLnO increases the logistic regression classifier’s capacity to discriminate between functional and defective states, resulting in improved maintenance scheduling and less downtime in manufacturing processes.
- Outlines potential future directions and avenues for exploration in subsequent research endeavors within the scope of the presented work.

### 1.4. Related Work

A theoretical frame of reference was developed by the Institute of Asset Management (IAM) to systematically control the management of physical, virtual, and human assets. This structure comprises six pillars: risk assessment, organization and people, asset information, life cycle delivery, strategy and planning, and asset management decision-making [30]. An innovative asset classification technique focusing on multiunit systems was proposed by the researchers. This technique segregates assets into fleets and portfolios based on the degree of homogeneity or heterogeneity [31]. It mainly targets the dependencies within such complex systems in terms of resource allocation, performance, and also stochastic consequences.

To enhance safety and reliability in multi-units systems, more refined models, which include multi-criteria and multi-dimensional decisions, have been developed. The MinRE technique of service placement has been developed to optimize quality of service (QoS)-influenced IoT devices with reduced energy consumption in fog computing. The study shows that the technique was better in simulated testing, as compared to cloud-only, edge-ward, and resource-aware approaches, by identifying services as critical or normal with respect to deadlines and service requirements [32]. Researchers suggested a method to optimize denial of service (DoS) attacks in IoT networks using the Teaching-Learning-Based Optimization (TLBO) algorithm and received signal strength (RSS) to diminish the severity of the attacks. This method found potential attackers with a false warning rate of 0.7% and with an accuracy of 12 cm using RSS [33]. This is load-balancing through intelligent industrial resource management with the use of the Jena architecture and the Contract-Net Protocol. The architecture combines a resource ontology model, Jena reasoning, and CNP-based procedures to maximize the effectiveness of resource allocation, as mentioned in the source [34]. They proposed a new task-scheduling mechanism based on threshold evaluation, arguing that containerization is much more effective and light-weighted than virtual machines (VMs) in fog computing. The simulation results revealed that containers outperformed virtual machines, but the computation performance and appropriateness of cloud resources were not considered [35].

In order to monitor industrial equipment, a lightweight hybrid architecture system called SERENA was designed. Machine-learning algorithms, including decision trees, gradient-boosted trees, and random forests, were used with SERENA, which combined cloud and edge computing, using sensor-collected data, processed in hybrid cloud environments, and delivered via load balancing mechanisms based on Docker [36].

IoT-based intelligent manufacturing is driven by seven key aspects, according to Rajnoha and Hadac [37]. These include data analytics, predictive analysis, process management, intelligent planning, visualization, and data security. Further research is necessary to determine how well these factors correlate with new IoT manufacturing technologies, particularly edge-fog computing solutions, even if they greatly increase industrial stability and productivity. Brous and Janssen [38] did a thorough assessment to assess the advantages and limitations of IoT implementation in commercial settings. Six organizational conditions and their implications were outlined in their research, along with benefits including automated decision-making and cost reduction. Nevertheless, although the study concentrated on the strategic deployment of IoT, it did not investigate edge-fog computing.

Hamm, Willner, and Schieferdecker [39] carried out an exploratory content study of current projects and suggested a roadmap that links the issues of edge computing with aspects of sustainable development. Their study did not investigate the possibility of creating profit using edge computing while identifying risks and opportunities. Mouradian et al. [40] performed a thorough analysis of fog computing, separating it from related ideas, assessing current algorithms and architectures, and outlining applications in content delivery networks and the IOT. In addition, it addressed present issues and potential avenues for future study by outlining important evaluation standards for fog-based systems. Using IoT-based embedded devices for remote tracking and water quality control, the study [41] investigates automated aquarium monitoring. With a focus on specialized routing protocols like IHELBRP, the study [42] examines UWSN issues. While evaluating their benefits and drawbacks, it looks at energy use, packet delivery speeds, and security issues.

The Attribute-Attention LSTM model recorded good performance at an accuracy of 84.60%, high precision at 89.79%, and recall at 94.43%. This model reflects the appropriateness of feature-focused temporal analysis [43]. Genetic algorithm-based resource scheduling combined with two-class logistic regression improved the performance result by 94.50% efficiency, along with balanced precision-recall, i.e., 94.60% and 93.30%, respectively [44]. In fact, some more hybrid complicated combinations, such as multi-scale dilation attention CNN with a probabilistic beetle swarm–butterfly optimizer, achieved very high results (that is, 95.91% accuracy and a 96% F1 score) on engine-related data sets, highlighting the potential of biologically inspired optimizers [45]. Knowledge graph-enhanced kNN-LSTM models also showed a robust result (91.09% accuracy and 96.30% recall) [46], while CNN–bidirectional LSTM frameworks were consistent in their precision-recall trade-offs (0.89–0.96) [47]. Simpler ensemble methods such as AdaBoost provide a very good outcome against individual learning methods (92%–precision and a 91% F1 score), which illustrate the power of classical algorithms for different applications in failure prediction [48]. Maintenance through Industry 4.0 technologies is valuable and really works towards downtime reduction and maximization of efficiency; however, challenges such as missing skilled labor and uncertainty with ROI still exist. No previous study has looked at an integrating form of beauty for such technology with maintenance incorporated. Eleven methods to come up with a uniform deployment model were analyzed in this research [49].

In Section 1, the document talks about Industry 4.0 and predictive maintenance, followed by a description of the challenges in asset management and how optimization algorithms such as GWO and J-SLnO play their respective roles. Section 2 gives the materials and methods such as the system model, algorithmic frameworks, and predictive maintenance workflow. Section 3 then presents the results in terms of comparing performance indices, including runtime, cost, energy use, and accuracy. As Section 4 discusses the findings, it includes the notable benefits of the proposed algorithms. Section 5 concludes with its important takeaways, limitations, and future research avenues.

## 2. Materials and Methods

### 2.1. System Model

**Figure 1:**
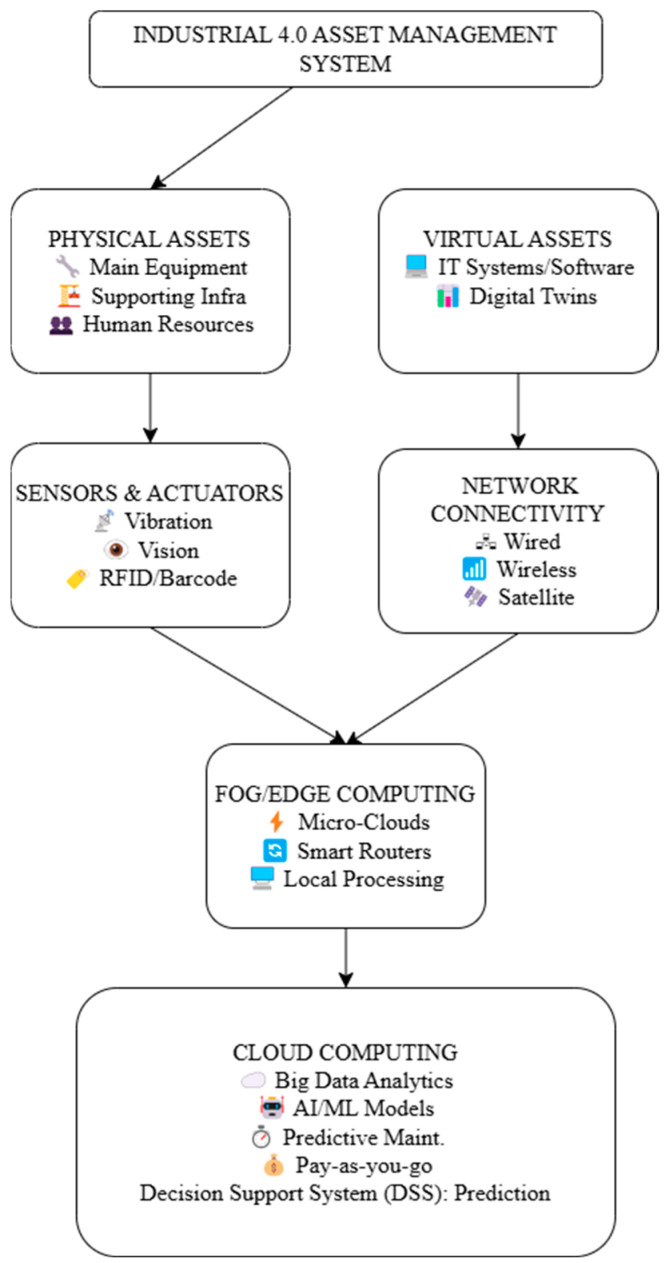
Proposed methodology.

The five core levels of the asset management system model—asset, perception, network, fog computing, and cloud computing—each have different functions and are shown in Figure 1.

All company resources that have economic worth and add to value generation are included in the asset layer. This includes four categories of assets: human, virtual, supporting, and primary physical assets. Primary physical assets, which include necessary gear and equipment, are the foundation of automation and manufacturing. The efficient operation and maintenance of major manufacturing processes are guaranteed by supporting physical assets. By integrating IT software into business and industrial processes, virtual assets contribute to digital transformation. Employees, suppliers, consumers, and end users are examples of human assets that actively participate in different phases of a product’s life cycle.

For manual duties, maintenance, and troubleshooting that automated systems cannot handle, employees are especially important. Industrial smart sensors that collect data about the environment and products are integrated into the perception layer. Predictive maintenance is aided via sensors integrated into equipment that track physical data. Vision sensors also make it easier to read barcodes and QR codes, which provide important asset-related information like kind, location, and purchase date. Furthermore, by controlling employee access and identifying time fraud, facial recognition technology improves human resource management. Data transportation from sensors to different parts, such as network infrastructure and fog computing, is made smooth through the network layer. It facilitates real-time communication via wired and wireless connections, business intranet networks, and satellite linkages, allowing manufacturing facilities all over the world to be connected. Decentralized data processing is made possible through fog computing, which sits between edge devices and cloud centers. Real-time asset analytics is made possible by handling data locally, which reduces latency and bandwidth consumption in comparison to cloud-based processing. Cloudlets, micro-clouds, application servers for industry-specific software, smart switches for facility management, and routers for Industrial Internet of Things (IIoT) applications are all part of this layer. IIoT-related jobs and industrial big data management occur at the cloud computing layer. Pre-processing of the data, model training, testing, forecasting, and deployment are all included in this. Pay-as-you-go solutions that comply with corporate standards enable organizations to optimize operations through the scalable and flexible resource management that cloud computing provides.

### 2.2. GWO

Gray wolf cooperative hunting behavior serves as the paradigm for the GWO, a metaheuristic algorithm that makes decisions using a hierarchical leadership structure. Within a wolf pack, the α wolf holds the highest rank, orchestrating the pack’s actions and dynamics. Despite not being the strongest physically, the α wolf possesses superior management skills. The β wolf ranks next, supporting the α wolf and acting as an intermediary between the alpha and other wolves. Further down the hierarchy are the Δ and ω wolves, each with diminishing authority. The Δ wolf assists the higher-ranked wolves and maintains the third-best command, while the ω wolf holds the lowest rank, following orders from the higher-ranked wolves. The Grey Wolf Optimiser (GWO) algorithm assigns wolves hierarchical roles depending on their fitness ratings. The best solution is labeled as α, the second-best as β, the third-best as Δ, and the remainder as ω. These responsibilities direct the optimization process via four stages: finding prey (exploration), encircling (convergence), attacking, and hunting (exploitation). The method starts by randomly initializing wolf placements; then, each iteration updates the hierarchy based on fitness values to improve the search for the best option [50,51].

The encircling behavior in the GWO algorithm mimics how wolves position themselves relative to their prey during optimization. The prey’s location at a particular iteration is designated as $\vec{X_{p}}\left(t\right)$ , while the wolves’ positions are represented as $\vec{X}\;(t)$ . The algorithm’s encircling action can be expressed mathematically using the following equation.

(1) $$\vec{D}=\left|\vec{C}\;.\vec{X_{p}}\left(t\right)-\vec{X}\;(t)\right|,$$

(2) $$\vec{X}\;\left(t+1\right)=\vec{X_{p}}\left(t\right)-\vec{A}.\vec{D},$$

where, t = iteration number, $\;\vec{A}\;and\;\vec{C}$ = bootstrap program coefficient vectors, $\vec{X_{p}}\left(t\right)$ = the position vector of the prey, $\;\vec{X}\;(t)$ = the position vector of a gray wolf, and $\vec{D}$ = the computation of vector to denote the updated location of the gray wolf.

(3) $$\vec{A}=2\vec{a}.\vec{r_{1}}-\vec{a},$$

(4) $$\vec{C}=2\vec{r_{2}},$$

$\vec{a}$ = The linear reduction in the vector set from 0 to 2 during the iteration.

$\vec{r_{1}}$ and $\vec{r_{2}}$ = the Optimizationin [0, 1].

**Figure 2:**
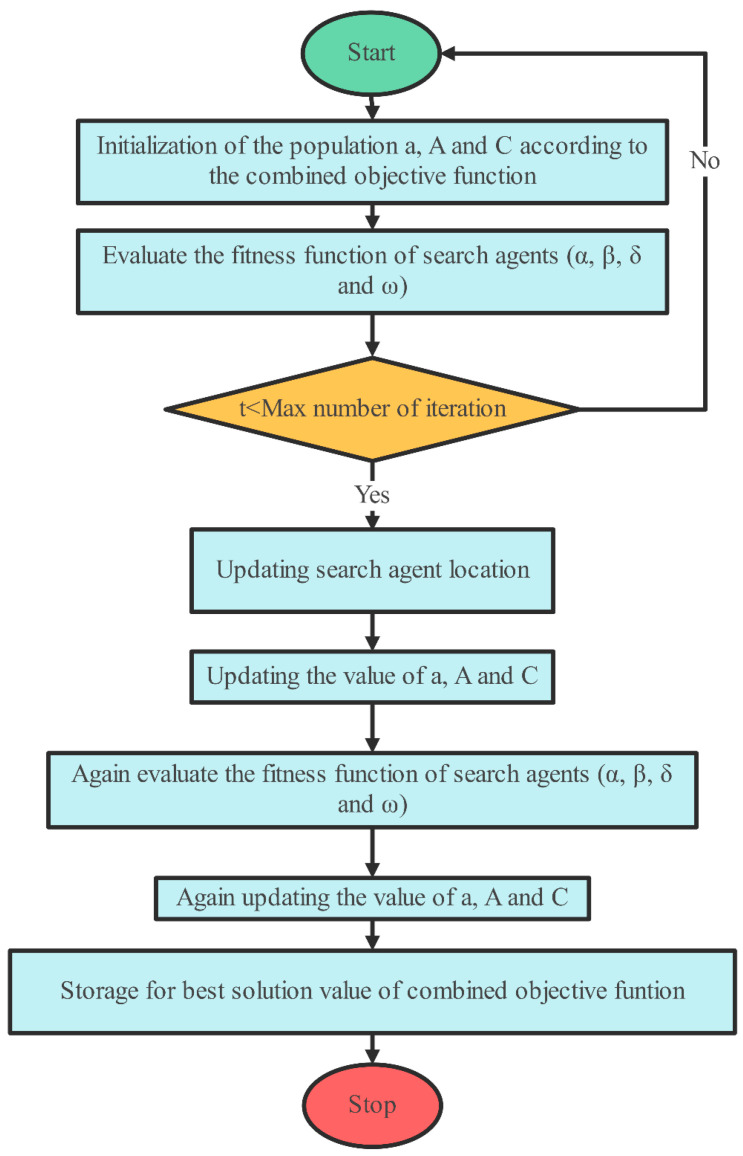
Flow diagram of GWO.

In a GWO algorithm, the location of the prey influences gray wolf movement. Each wolf alters its location (x, y) in response to the prey’s placement (x′, y′), resulting in an adaptive search process. This movement is governed by two parameters: A, which governs the step size and exploration–exploitation balance, and C, which provides randomization to boost search variety. This dynamic placement allows the algorithm to efficiently converge on the best option. The hunting behavior of the wolves continues until the prey halts its movement, emphasizing persistent attacking actions. Throughout the algorithm’s simulation, the value of parameter a decreases, while the variation rate A also diminishes. The α, β, and δ agents retain info regarding the prey’s position. These three best solutions guide the algorithm’s process, with the ω then introduced as the 4th agent to refine its spot within the search area. α, β, and δ agents estimate the prey’s site, orchestrating the positioning of wolves around the prey, which is regulated by the ω. The procedure of the GWO algorithm is depicted in Figure 2 and Algorithm 1, illustrating the algorithm’s flow diagram and pseudo-code, respectively. The GWO model’s formulation is described through the following equations.

(5) $$\vec{D_{\alpha }}=\left|\vec{C}\;.\vec{X_{\alpha }}-\vec{X}\;\right|$$

(6) $$\vec{D_{\beta }}=\left|\vec{C}\;.\vec{X_{\beta }}-\vec{X}\;\right|$$

(7) $$\vec{D_{\delta }}=\left|\vec{C}\;.\vec{X_{\delta }}-\vec{X}\;\right|$$

(8) $$\vec{X_{1}}=\left|\vec{X_{\delta }}-A_{1}(\vec{D_{\alpha }})\;\right|$$

(9) $$\vec{X_{2}}=\left|\vec{X_{\beta }}-A_{2}(\vec{D_{\beta }})\;\right|$$

(10) $$\vec{X_{3}}=\left|\vec{X_{\delta }}-A_{3}(\vec{D_{\delta }})\;\right|$$

(11) $$\vec{X}\;\left(t+1\right)=\frac{\vec{X_{1}}+\vec{X_{2}}+\vec{X_{3}}}{3}$$

The GWO technique offers several characteristics that make it suitable for addressing resource scheduling problems:

- Global search: GWO initializes its search from multiple points across the population, aiding in comprehensive exploration, rather than focusing on a single point, and making it conducive for global search in complex solution spaces.
- Avoidance of local optima: by using a global search strategy, the algorithm avoids being trapped in local optima, which may otherwise restrict the search to less-than-ideal answers.
- Efficient exploration: the GWO method is ideally suited to efficiently addressing large-scale optimization problems because it efficiently searches the solution space.
- Flexibility and adaptability: the algorithm’s inherent adaptability allows it to handle diverse optimization problems, making it applicable to different resource scheduling scenarios.
- Performance: for resource-scheduling jobs with high computing needs, GWO is a great option since it usually performs well in terms of both convergence speed and accuracy.

These characteristics collectively position GWO as a promising approach to resource scheduling, particularly in scenarios where finding optimal or near-optimal solutions across a vast solution space is essential.

**Algorithm 1: GWO-based resource scheduling technique**
**Input: Size of problem, size of population** **Output: scheduling decision*****1:*** ***2:*** ***3:*** ***4:*** ***5:*** ***6:*** ***7:*** ***8:*** ***9:*** ***10:*** ***11:*** ***12:*** ***13:*** ***14:*** ***15:*** ***16:*** ***17:***Start Set the gray wolves population, X_i_ (i = 1, 2, …, n) Based on the combined goal function, set the starting values for variables a, A, and C. Determine which search agents are most suitable by assessing their fitness. The best answer among the search agents is represented by X_α_, the second-best by X_β_, and the third-best by X_δ_ among the search agents. Set t at 0 While (t < highest iteration limit) For each and every search agent Adjust the current search spot based on the provided equation End for Change a, A, and C’s values to reflect the integrated objective function. Recalculate the fitness values for each search agent and categorize them according to their performance. Once again, modify the locations of X_α_, X_β_, and X_δ_. Now t is t + 1 Store the value of the ideal solution. End while End

### 2.3. JA Optimization

The principle of the JA method is presented via performance assessment using an unlimited baseline sphere function [52]. In this context, the variable count at any iteration, denoted as ‘a’, covers the set of design variables, indexed as ‘j’ from 1 to ‘a’. Similarly, the population size ‘b’, representing the count of candidate solutions, is indexed as ‘c’ from 1 to ‘b’. Within this framework, the terms ‘best’ and ‘worst’ designate the candidate solutions that yield the highest and lowest function values, respectively. During the it^th^ iteration, the bth variable for the eth candidate is represented as X_j, c, it_ and undergoes modification utilizing Equation (12). Random numbers rnd1_j,it_ and rnd2_j,it_ within the range [0, 1] influence this flexible alteration. The jth variables of the worst and greatest choices are represented as X_j,worst,it_ and Xj_,best,it,_ respectively.

The method reduces the sphere function, aiming for an ideal solution of zero within the range [−100, 100]. The stepwise procedure for the conventional JA algorithm is delineated in Algorithm 2.

(12) $$X_{j,c,it}^{\prime }=X_{j,c,it}+{rnd1}_{j,it}\left(X_{j,best,it}-\left|X_{j,c,it}\right|\right)-{rnd2}_{j,it}\left(X_{j,worst,it}-\left|X_{j,c,it}\right|\right),$$

The pseudocode of the Jaya Algorithm (JA) describes a relatively simple yet effective optimization procedure in which solutions are iteratively improved using the best and worst candidates in the search space. It is based on defining perfect and worst solutions (lines 1–2) and updating candidate solutions according to Equation (13), which refers to both best and worst locations for explorative and exploitative updating (lines 3–6). It evaluates the updated solution if it is better than the best so far (line 7), keeps the good ones, and removes the bad ones (lines 8–10). This procedure continues until it reaches an iteration limit or meets other termination criteria (lines 11–15). The Jaya Algorithm is parameter-free, solely relying on the relative fitness of solutions for convergence without complex tuning. Thus, it realizes dynamic balance attraction toward the best solution and repulsion from the worst, giving the algorithm efficient path traversal through the search space and making it broadly applicable to different optimization problems yet computationally light. JA’s pseudocode illustrates the flexibility and robustness of this algorithm: however, the performance will obviously differ with respect to specific features of the problem and the landscape of its objective functions.
**Algorithm 2: Pseudocode of conventional JA** [52]***1:*** ***2:*** ***3:*** ***4:*** ***5:*** ***6:*** ***7:*** ***8:*** ***9:*** ***10:*** ***11:*** ***12:*** ***13;*** ***14:*** ***15:***Identify the optimal and least favorable outcomes Adjust the solutions by incorporating Equation (13), considering both optimal and worst solutions as benchmarks. If 
$\left(X_{j,best,it}^{\prime }\;is\;better\;than\;X_{j,best,it}\right)$
 (13) While (iteration < maximum iteration limit) Revise the resolutions by incorporating the formulations outlined in Equation (13) Take and replace the current solution Else Store the earlier solution End if If (termination criteria is met) Provide the best possible solution. Else Store best and worst options End

### 2.4. Standard SLnO Algorithm

The hunting habits of sea lions in their vast colonies serve as the model for the conventional SLnO algorithm. These animals may move among these subgroups for hunting because they establish hierarchical groupings according to sex, age, and activity. A lead lion usually locates prey and calls for other lions to join the hunt. By considering the target prey to be the best option, the algorithm imitates this procedure. A random vector (rad), the gap between the sea lion and the prey (dst), and particular locations (tr(it) and X(it)) all affect how the sea lions travel in the direction of the prey in the computational framework (Equation (14)) of SLnO. In subsequent iterations (Equation (15)), the sea lions progressively approach the prey, guided by their encircling behavior by a constant term (C) that diminishes with time [53].

(14) $$dst=|2rad.tr\left(i\right)-X\left(it\right)|,$$

(15) $$X\left(it+1\right)=tr\left(it\right)-dst.C,$$

(16) $${SS}_{ldr}=|\frac{\left({ss}_{1}(1+{ss}_{2}\right)}{{ss}_{2}}|,$$

(17) $${ss}_{1}=\sin\theta ,$$

(18) $${ss}_{2}=\sin\theta ,$$

Sea lions’ communication during hunting, both in water and on the shore, involves distinct sounds, serving as signals to coordinate their actions. The algorithm’s mathematical representation (Equation (16)) embodies this communication behavior, reflecting the leader’s call’s sound speed (SS_1dr_) and the differential speed of sound in air (ss_1_) and water (ss_2_), calculated separately in Equations (17) and (18), respectively. These equations encapsulate the adaptation of sea lion communication dynamics into the optimization process, simulating their ability to recognize and encircle prey.

(19) $$X\left(it+1\right)=\left|tr\left(it\right)-X\left(it\right)\right|.\cos\left(2\pi rad\right)+tr(it),$$

(20) $$dst=|2rad.X_{rand}\left(it\right)-X(it)|,$$

(21) $$X\left(it+1\right)=X_{rand}\left(it\right)-dst.A,$$

During the hunting phase, sea lions exhibit a collective strategy to locate and surround their prey. The leader takes charge by identifying the target and communicating its position to others. Often, the prime objective for the group is the current best solution, mimicking the sea lions’ targeting of prey in the wild. This hunting behavior is represented mathematically through a process known as the ‘dwindling encircling mechanism; and ‘circle updating position’. The encircling process is driven by a parameter C, defined in Equation (19), determining the approach sea lions take to surround the prey. Their movement, akin to chasing a school of fish, initiates from the edges toward the center. The gap between each sea lion’s position and the best solution is designed using |tr(it) − X(it)|, where || denotes absolute value, and rad introduces randomization between −1 and 1. This randomness emulates the zigzag motion of sea lions’ whiskers as they search for prey. When C < 1 or is negative, sea lions shift their location away from the leader and target to follow the most effective search agent. Conversely, if C exceeds 1, the SLnO algorithm integrates global search agents to identify the overall optimal solution, employing Equations (20) and (21) [53].

Here, X_rand_(it) stands for a sea lion that was chosen at random from the existing population. Algorithm 3 contains the pseudocode for the conventional SLnO method.
**Algorithm 3: SLnO Algorithm** [53]***1:*** ***2:*** ***3:*** ***4:*** ***5:*** ***6:*** ***7:*** ***8:*** ***9:*** ***10:*** ***11:*** ***12:*** ***13:*** ***14:*** ***15:*** ***16:*** ***17:*** ***18:*** ***19:***Start Initialized the population Choose X_rand_ For every search agent, determine its fitness function. The search agent that performs the best and has the greatest fitness level is the X. while (t < total number of iterations) Equation 
${SS}_{ldr}=|\frac{\left({ss}_{1}(1+{ss}_{2}\right)}{{ss}_{2}}|$
, to compute SS_ldr_. if (SS_ldr_ < 0.25) If (|C| < 1) Using dst =|2rad.tr(i) − X(it)|, the current search agent’s spot is updated. Else Select an arbitrary search agent (X_rand_) Use 
$X\left(it+1\right)=X_{rand}\left(it\right)-dst.A$
, to modify the position of the active search agent. Else To improve the present search agent’s position, use Equation 
$X\left(it+1\right)=\left|tr\left(it\right)-X\left(it\right)\right|.\cos\left(2\pi rad\right)+tr(it)$
. Identify the fitness function of each search agent. If a better solution is available, update X. Return X as the optimal solution. End while

### 2.5. J-SLnO Algorithm

**Figure 3:**
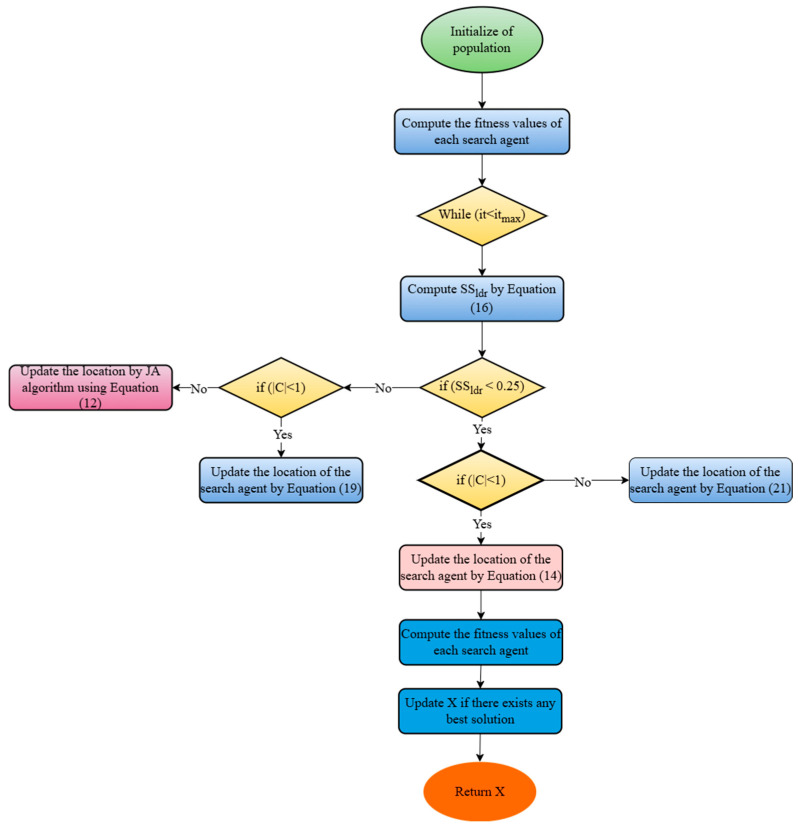
Flowchart of planned J-SLnO algorithm.

The SLnO algorithm is renowned for its effective exploration and strong performance in standard functions, and it was inspired by the hunting habits of sea lions. It often becomes stuck in local optima, however. We have combined the SLnO approach with the Jaya algorithm to increase its efficacy, with the goals of maximizing accuracy, decreasing computation time, and optimizing control parameters. The update procedure in the recently suggested J-SLnO method is condition-dependent; the standard update takes place when the sea lion leader’s vocalization speed falls below 0.25 (SS_ldr_ < 0.25). Equation (21) is used to update whether the requirement (|C| < 1) is satisfied. In any other situation, the update adheres to the Jaya algorithm’s instructions (Equation (12)). By fusing the best features of many optimization approaches, this hybrid model has shown enhanced performance in resolving a range of search issues, especially with quicker convergence [54]. Figure 3 displays the flowchart for the J-SLnO method, which is further explained in Algorithm 4.

**Algorithm 4: J-SLnO Algorithm.**
***1:*** ***2:*** ***3:*** ***4:*** ***5:*** ***6:*** ***7:*** ***8:*** ***9:*** ***10:*** ***11:*** ***12:*** ***13:*** ***14:*** ***15:*** ***16:*** ***17:*** ***18:*** ***19:*** ***20:*** ***21:*** ***22:***Start Initialized the population Choose X_rand_ For every search agent, determine its fitness function. The most suited potential search agent is the X. while (t < total number of iterations) Equation (16) to compute SS_ldr_. if (SS_ldr_ < 0.25) If (|C| < 1) Equation (14) is used to update the position of the active search agent. Else Select an arbitrary search agent (X_rand_) Equation (21) should be used to update the position of the active search agent. Else if (|C|< 1) Adjust the current search agent’s location by using Equation (19). Else Using the Jaya technique and the calculation given in Equation (12), update the position. Determine each search agent’s fitness function. If a better solution is available, update X. Return X as the top solution end while

The J-SLnO technique exhibits several characteristics that make it suitable for solving resource scheduling problems:

- Exploration and exploitation: J-SLnO combines the exploration ability of Jaya optimization with the exploitation capability of Sea Lion Optimization. This hybridization facilitates a balanced exploration of the search space while efficiently exploiting promising regions, aiding in better convergence.
- Convergence speed: J-SLnO is known for its faster convergence rates, allowing it to reach near-optimal solutions within a relatively shorter number of iterations related to other optimization algorithms.
- Adaptability and flexibility: the hybrid nature of J-SLnO offers adaptability to diverse optimization problems, including resource scheduling scenarios with varying constraints and objectives.
- Global search capability: the synergy between Jaya optimization and Sea Lion Optimization provides J-SLnO with the ability to perform global searches effectively, exploring a wide solution space to avoid local optima.
- Efficient population-based approach: leveraging a population-based approach, J-SLnO can simultaneously maintain multiple potential solutions, enabling diverse exploration and aiding in escaping from suboptimal solutions.
- Robustness and stability: J-SLnO tends to exhibit robust performance by balancing exploitation and exploration, providing stable and consistent convergence behavior across different problem instances.
- Parallelism and scalability: its parallel nature allows for the exploration of multiple solutions simultaneously, making it scalable for complex resource scheduling scenarios with large-scale optimization requirements.
- Competitive performance: J-SLnO often demonstrates competitive performance in terms of convergence speed, accuracy, and the ability to handle multi-objective or constrained optimization problems, making it suitable for resource scheduling tasks where multiple factors need to be considered.

Overall, the hybridization of Jaya optimization with sea lion optimization in J-SLnO offers a promising approach for resource scheduling problems, emphasizing faster convergence, exploration–exploitation balance, adaptability, and competitive performance in finding optimal or near-optimal solutions.

### 2.6. Manufacturing Equipment Predictive Maintenance

In the manufacturing sector, machinery like die casting, laser cutting, and plasma cutting devices plays a pivotal role in producing goods for customers. However, unexpected machine breakdowns and component failures can halt production lines, causing a ripple effect. These unplanned stoppages result in delivery delays, disrupt industrial processes, and lead to financial setbacks.

Predictive maintenance in Industry 4.0 use IIoT sensors to gather and analyze real-time industrial data, allowing for early diagnosis of equipment problems. This strategy improves ‘Overall Equipment Efficiency’ (OEE) by continuously monitoring equipment health, decreasing downtime, and enhancing manufacturing performance via data-driven maintenance programs.

This case study was conducted on a computer with the following specifications: processor: 11th Gen Intel Core i5-1135G7; CPU@ 2.40 GHz, RAM: 64 GB; operating system: Windows 11, 64-bit.

### 2.7. Data Set

Fidan Boylu Uz [55] created the data sets that were used in this particular case study. The attribute data were provided in a broad manner with both words and numerical numbers in order to protect intellectual property and company secrets. They consist of a total of 291,669 samples, with the remaining 285,006 samples (97.72%) being classed into class ‘0’ for non-malfunctioning equipment, while 6663 samples (2.28%) were classed into class ‘1’ for malfunctioning equipment. This extreme imbalance in the data set will pose challenges for model training, in that the machine-learning model may be skewed toward the majorityclass and become highly accurate but poorly generalized at detecting malfunctions.

#### 2.7.1. Data Set Description

The data set includes eleven attributes essential for providing information regarding the performance of industrial equipment. These attributes are as follows:

‘datetime’ denotes the period for the collection of data (convenient date—time).

‘machineID’—the respective machine is identified by its unique ID.

‘errorID’ shows an error code corresponding to the respective type of issues confronted.

‘voltage’—electrical voltage in volts.

‘rotate’—speed of rotation of the machines.

‘pressure’—pressure reading from the equipment.

‘vibration’—level of vibration seen in the equipment.

‘comp’—the change or replacement of components in the equipment.

‘model’—equipment type/model.

‘age’—age of the equipment, which possibly relates to performance.

‘failure’—the indicator of failure categorized into binary values: 1 for an incidence of malfunction and 0 for a non-incidence of malfunction.

The measurement of different variables might be numerical and categorical, giving diversity to the representation of the operational state of industrial equipment. Hence, it indicates that ‘errorID’, ’model,’ etc., are categorical variables, and certain encoding techniques might be used prior to fitting in a particular machine-learning model. Moreover, ‘datetime’ may require feature engineering to derive worthwhile time-based patterns.

#### 2.7.2. Preprocessing and Management of Class Imbalance

The preprocessing of this data set was characterized by one of the most important aspects, tending to a serious class imbalance. Since only 2.28 percent of the total number was labeled class ‘1’ (malfunctioning), the data set was highly skewed towards the majority class (class ‘0’). A machine-learning model, if trained on a heavily imbalanced dataset, will make biased predictions by favoring the majority class and thus reducing its ability to identify failures correctly.

In this situation, an under-sampling approach was adopted. Under-sampling is a method of decreasing the number of majority-class samples to match or be nearer to minority-class samples. Thus, by achieving an equal distribution of classes, it helps balance the data set and allows for an effective pattern that the model will learn from both classes.

The process of under-sampling was performed in the following stages:

A set of majority-class samples was randomly selected—given 285,006 samples in class ‘0’, some instances were removed in order to achieve balance in the data set.

Matching the sample closer to that of the minority class—the data set was manipulated in such a way that 6663 samples were left of class ‘1’ and 7819 of class ‘0’, thus giving a total number of 14,482 samples in the final processed data set.

Assuring representative selection—the under-sampling strategy preserved a good diversity of ‘0’ class instances to maintain meaningful variability in the data set.

This preprocessing step greatly improved class distribution for the data set to ensure that the machine-learning model was learning from performance-related patterns of both failure and non-failure instances and not heavily induced via the majority class. The downside is that under-sampling leads to a potential loss of useful information since a number of samples from the majority class are discarded. Under-sampling was applied to further balance the data set, making it suitable for any predictive modeling activity. By reworking the dataset to contain 14,482 samples with a much more equal distribution between failure and no-failure instances, a very usable machine-learning model was made for classifying this dataset without overwhelming any bias in favor of the majority class.

### 2.8. Employment of the Proposed Techniques in Fog Work Flow Sim

Four distinct end device types—voltage, pressure, vibrational, and rotational sensors—were simulated using the Fog Work Flow Sim framework. In addition, it has a cloud server and 5 fog nodes: the cloudlet, application server, smart switch, micro-cloud, and smart router. Each device’s computing power (measured in MIPS values) and execution costs adhere to the guidelines given in [56]. The configuration settings for the Fog Work Flow Sim are detailed in Table 1. The parameters chosen for optimization algorithms, specifically the cross rate and mutation rate, were carefully adjusted based on the specific problem under consideration. The meticulously modified parameters, which were taken from earlier research [56,57,58,59], are shown in Table 2 with the aim of improving many performance indicators, including time, energy consumption, and overall cost. The evaluation of performance was conducted using Equations (22)–(27) [60,61].

Execution Time

(22) $$t_{t}^{total}=t_{t}^{tran}+t_{t}^{exe}+t_{t}^{rec}+t_{t}^{mig},$$

Cost

(23) $$C_{t}^{total}=T_{i}^{fog}\times C^{fog}+T_{i}^{cloud}\times C^{cloud},$$

Energy usage,

(24) $$E_{i}^{total}=E_{i}^{x}+E_{i}^{y}+E_{i}^{z},$$

(25) $$E_{i}^{x}=\frac{Data\;transmission}{Bandwidth}\times P_{transmission},$$

(26) $$E_{i}^{y}=\frac{Task\;workload}{Task\;processing\;speed}\times P_{idle},$$

(27) $$E_{i}^{z}=\frac{Task\;workload}{Task\;processing\;speed}\times P_{end},$$

Both the processing time at the server and the data transmission time from IoT devices to the fog server are $t_{i}^{exe}$ and $t_{i}^{tran}$ , respectively. The transmission time between the fog server and the end device is denoted as $t_{i}^{rec}$ , whereas the time required for a task to transfer to a new fog server as a result of the end device’s mobility is denoted as $t_{i}^{mig}$ .

The variable $t_{i}^{fog}$ denotes a workflow task allocated to the fog server, while $t_{i}^{cloud}$ represents a workflow task allocated to the cloud server. *C*^*fog*^ signifies the cost per second for utilizing the fog server, and *C*^*cloud*^ represents the cost per second for utilizing cloud computing services.

$E_{i}^{x}$ represents the energy consumed during transmission from end devices to the fog server, while $E_{i}^{y}$ signifies the energy utilized by end devices during idle periods, and $E_{i}^{z}$ represents the energy utilized for load processing.

Scientific workflows aim to illustrate task interdependencies and oversee data flow management. The Montage workflow, consisting of 60 jobs, has demonstrated the capacity to optimize performance metrics.

### 2.9. Two-Class Logistic Regression for Predictive Maintenance

The equipment in this study is represented by a value of 0 while it is operating and by a value of 1 when it is malfunctioning. ErrorID, voltage, rotation, pressure, vibration, compression, and age are some of the variables from the data set that are thought to be important contributors to possible failures. To create and analyze the predictive model, the data set is divided into two subsets: the testing set, which is used to test the model’s accuracy using real-world data, and the training set, which is used to create the model. The two-class logistic regression’s adjusted parameters are described in detail in Table 3.

Logistic regression was chosen mainly because it is an interpretable, computationally efficient, and good discriminator for binary classifications such as equipment failure, as opposed to normal operation. Unlike complex ANN or SVM types of models from which it may be difficult to extract the logic, logistic regression has very transparent coefficients, which go well with the increasingly important requirement by industries for explainable models in predictive maintenance. The linearity assumption of logistics regression also fits well with the pre-processed data set, where feature engineering (such as under-sampling) ensured classes were linearly separable.

### 2.10. Performance Evaluation Metrics

AUC, MEP, SMAPE, RMSE, F1 score, accuracy, recall, precision, and other performance measures were used to assess the model’s effectiveness. J stands for fitted data points, i for incremental values for each point, Fv for forecast values, and Av for actual values.

(28) $$MEP=\frac{100\%}{j}\sum_{i=1}^{j}\frac{Av-Fv}{Av},$$

(29) $$SMAPE=\frac{100\%}{j}\sum_{i=1}^{j}\frac{\left|Fv-Av\right|}{\frac{\left(\left|Av\right|+\left|Fv\right|\right)}{2}},$$

(30) $$RMSE=\sqrt{\frac{\sum_{i=1}^{j}{\left({Av}_{i2}-{Fv}_{i1}\right)}^{2}}{j}},$$

TP = true positive.

TN = true negative.

FN = false negative.

FP = false positive.

The model’s performance was evaluated using Accuracy Equation (31), which calculates the ratio of correct predictions to total predictions.

(31) $$Accuracy=\frac{TP+TN}{TP+TN+FN+FP},$$

Precision. The percentage of properly predicted positive samples, which is expressed in the following equation, was used to evaluate the model’s accuracy.

(32) $$Precision=\frac{TP}{TP+FP},$$

Model recall, a measure of the model’s ability to accurately identify instances of the positive class, was calculated using Equation (33).

(33) $$Recall=\frac{TP}{TP+FN},$$

F1 score. The following formula, which represents the harmonic mean of a model’s accuracy and recall, was used to obtain the F1 score:

(34) $$F1\;Score=2\times \frac{\left(Recall\times precision\right)}{\left(Recall+precision\right)},$$

(35) $$MCC=\frac{\left\{\left(TP\times TN\right)-(FP\times FN)\right\}}{\sqrt{\left(TP+FP)(TP+FN)(TN+FP)(TN+FN\right)}}$$

An indicator of the area under the ROC curve is the AUC score. When the AUC score is 1.0, the classifier is considered flawless.

## 3. Results

### 3.1. Execution-Time Analysis

**Figure 4:**
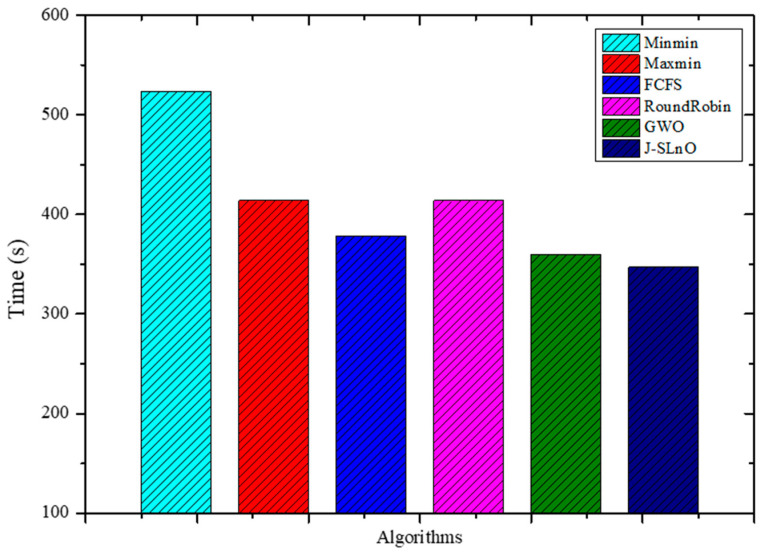
Evaluation results for various methods: time.

From Figure 4, GWO and J-SLnO were compared with other algorithms; GWO was faster than MinMin, MaxMin, FCFS, and RoundRobin, while J-SLnO was also faster than all the mentioned algorithms. GWO was about 31% faster than MinMin, 13% faster than MaxMin, around 5% faster than FCFS, and approximately 13% faster than RoundRobin. Similarly, J-SLnO was about 33% faster than MinMin, around 16% faster than MaxMin, nearly 8% faster than FCFS, and approximately 16% faster than RoundRobin. The comparison indicates that GWO and J-SLnO performed notably better in terms of time efficiency compared to the MinMin, MaxMin, FCFS, and RoundRobin algorithms, with GWO showing an advantage of around 13–31% faster performance, and J-SLnO demonstrating roughly 16–33% faster execution times across these algorithms.

### 3.2. Cost

**Figure 5:**
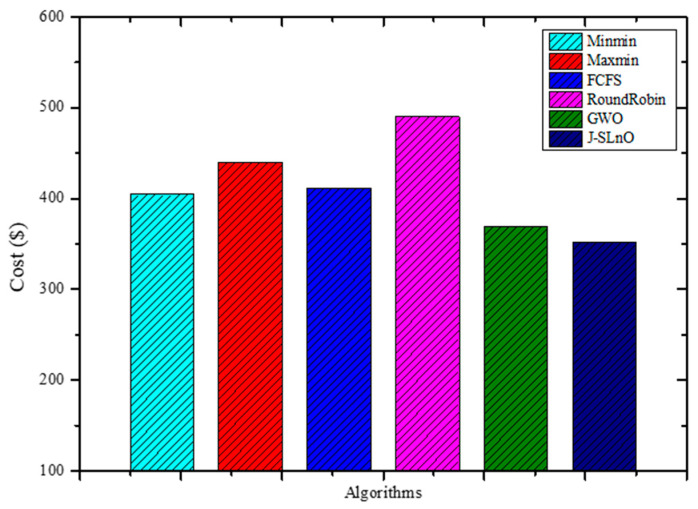
Evaluation results for various methods’ costs.

Figure 5 presents cost metrics associated with different algorithms. Among the listed algorithms, GWO (Grey Wolf Optimization) and J-SLnO (Jaya-based Sea Lion Optimization) showcase notably lower costs compared to MinMin, MaxMin, FCFS, and RoundRobin. GWO and J-SLnO demonstrate cost efficiencies at 369.13 and 352.24 dollars, respectively, while the other algorithms range between 404.53 and 490.25 dollars. In analyzing the cost differences, GWO stands out as approximately 8.57% cheaper than the most expensive algorithm, RoundRobin, and around 9.17% cheaper than MaxMin. Similarly, J-SLnO appears 13.56% cheaper than RoundRobin and approximately 19.71% cheaper than MaxMin. These relative cost benefits between J-SLnO and GWO indicate that they may be more affordable options among the algorithms on the list. Furthermore, both J-SLnO and GWO exhibit steady cost savings across a range of measures, demonstrating their capacity for optimization. According to our cost study, GWO and J-SLnO could be good options for situations when cutting costs is a top priority without sacrificing algorithm performance. Their reduced costs highlight their possible use in resource-constrained settings, which makes them attractive choices for optimization purposes is a key factor.

### 3.3. Energy Usage

**Figure 6:**
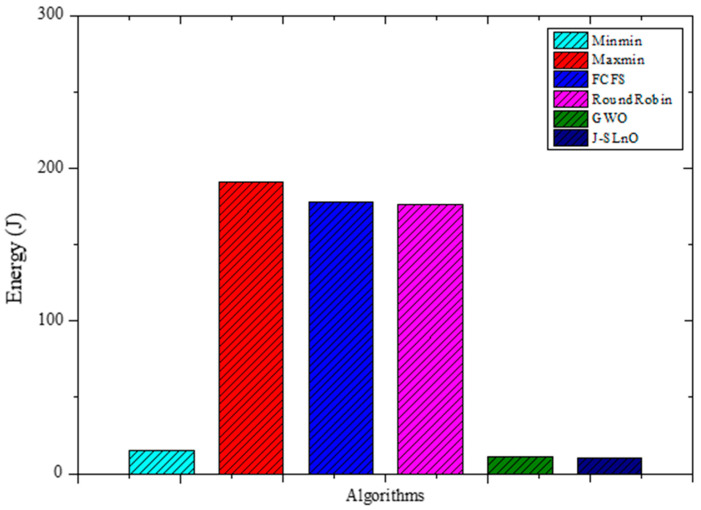
Evaluation results for MinMin, MaxMin, FCFS, RoundRobin, GWO, and J-SLno for Eergy.

The energy consumption of several algorithms is shown in Figure 6, which includes figures for MinMin, MaxMin, FCFS, RoundRobin, GWO, and J-SLnO. When compared to other algorithms, GWO and J-SLnO show much-reduced energy usage. There is a significant difference between GWO, J-SLnO, and the other algorithms when the difference in energy use is examined. GWO and J-SLnO showcase energy efficiency, consuming significantly less energy compared to MinMin, MaxMin, FCFS, and RoundRobin. When GWO and J-SLnO are compared to the average energy consumption of the other algorithms (MinMin, MaxMin, FCFS, and RoundRobin), GWO’s energy consumption is approximately 94.1% less than the average, while J-SLnO’s energy consumption is around 94.2% less. This stark contrast highlights the potential energy efficiency benefits offered by GWO and J-SLnO in algorithmic implementations, suggesting their viability in scenarios prioritizing energy conservation and cost reduction.

### 3.4. Confusion Matrix Analysis

#### 3.4.1. GWO Matrix Analysis

**Figure 7:**
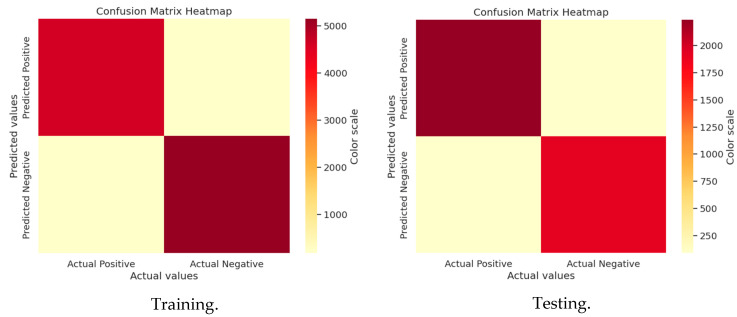
Confusion matrix of GWO training and testing.

A confusion matrix evaluates machine-learning model performance by categorizing predictions into true positives, true negatives, false positives, and false negatives, enabling the calculation of accuracy, precision, recall, and F1 score. It is useful since it provides information on a model’s advantages and disadvantages, assisting in determining what worked and what needs to be improved. Model performance may be improved by comprehending these specifics, guaranteeing improved accuracy and dependability for real-world use scenarios. Figure 7 and Figure 8 show the confusion matrices for GWO and J-SLno.

#### 3.4.2. J-SLno Confusion Matrix Analysis

**Figure 8:**
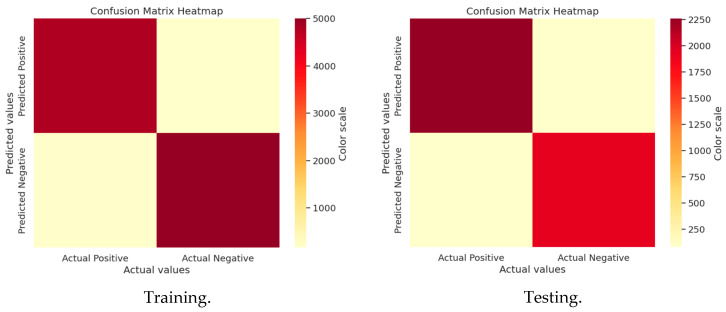
Confusion matrix of J-SLno training and testing.

### 3.5. ROC-AUC Analysis

**Figure 9:**
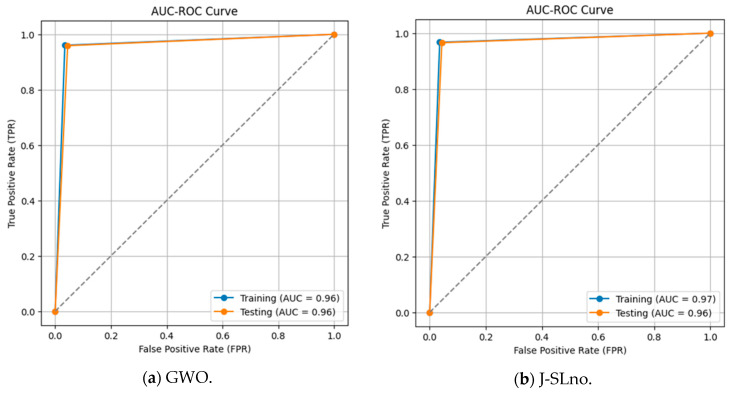
ROC-AUC curve.

The classifier’s ability to discriminate between positive and negative classes is shown in the ROC curves for the training and testing data sets (Figure 9). The AUC values are 0.96 for both data sets in one scenario and 0.97 (training) and 0.96 (testing) in another, with a constant threshold of 0.5. A better classification ability is indicated by a greater AUC, where 1 denotes flawless categorization. Strong model performance is shown in the findings, which show how well the model separates the two groups.

### 3.6. Accuracy Comparison

**Figure 10:**
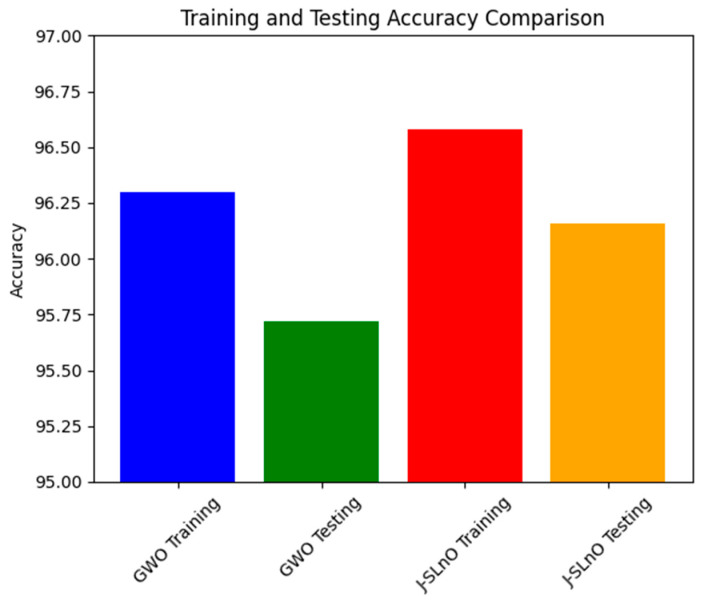
Accuracy analysis for various algorithms.

Figure 10 depicts the accuracy scores for both training and testing phases using two different algorithms, J-SLnO (Jaya-based Sea Lion Optimization), and GWO (Grey Wolf Optimization). These measurements show how well each predictive algorithm–façade works in terms of achieving a task. In this case, both algorithms showed overall good accuracy values during the testing and training stages except for J-SLnO, which shows somewhat better accuracy than GWO, recording scores of 96.58% during training and 96.16% during testing whereas GWO yielded training and testing scores of 96.3% and 95.72%, respectively. The importance of these numbers lies in understanding the robustness and generalization capabilities of the algorithms. The fact that both GWO and J-SLnO perform similarly through training and testing indicates that both algorithms can generalize well in the new data input situation. Thus, it is merely a small drop from training to testing expected and valid, so both algorithms will be slightly less efficient in fresh new data due to overfitting. Thus, both J-SLnO and GWO are efficient in learning from the data provided in the training phase and predicting accurately from unfamiliar, untested testing data. This minor performance difference between the algorithms indicates that perhaps J-SLnO could generalize fresh data slightly better than GWO.

### 3.7. Precision Comparison

**Figure 11:**
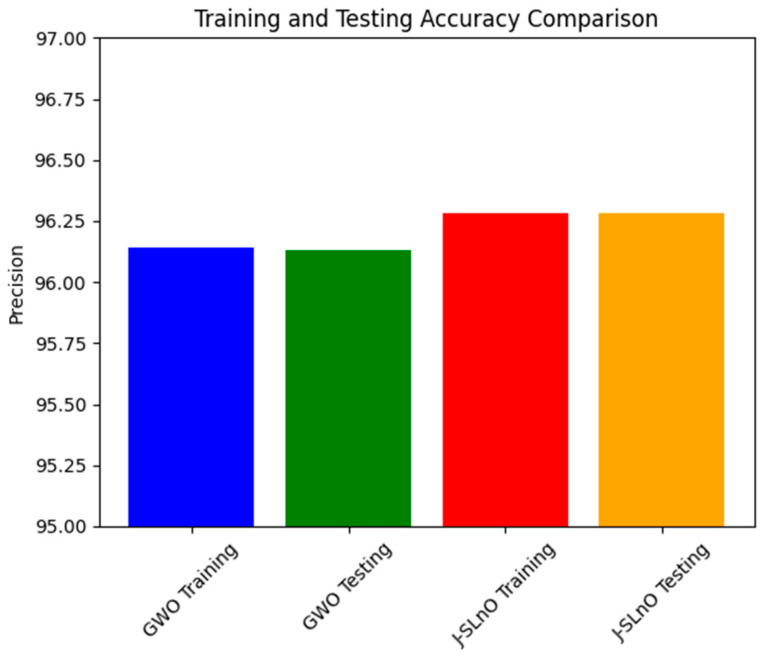
Precision analysis for various algorithms.

The accompanying Figure 11 gives the precision values achieved using GWO and J-SLnO Dispositions presented for the training and testing data sets. Precision is an important parameter to measure in relation to the classification model, as it determines how exactly the model makes positive predictions. In the training and testing conditions, GWO and J-SLnO achieve quite good precision values. While J-SLnO scores some more accuracies of 96.28% on both training and testing data sets, GWO scored a precision of 96.14% in training and 96.13% in testing. This number is significant since both optimization techniques consistently showed high accuracy values throughout training and testing periods.

These accuracy rates imply that, irrespective of the data set to which they are applied, both GWO and J-SLnO stand strong and dependable in producing maximal positive predictions. The algorithms seem to withstand prediction abilities when subjected to new unknown data, as viewed from the uniformity in accuracy score between training and testing data set. This consistency in precision establishes the models of GWO and J-SLnO as being stable and generalized, thus making them very applicable in real-life applications, which might be those demanding accurate positive predictions, such as in sentiment analysis, fraud detection, and medical diagnosis.

### 3.8. F1-Score Comparison

**Figure 12:**
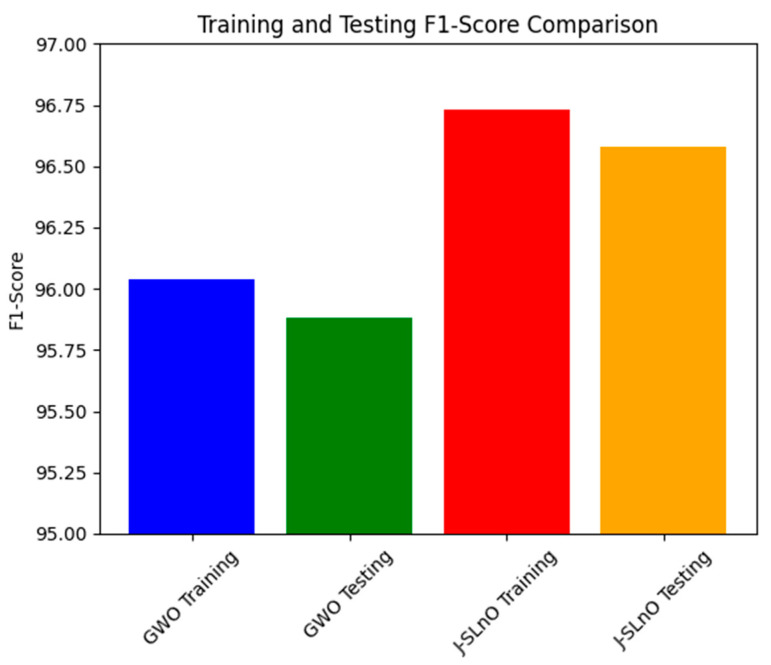
F1-score analysis for various algorithms.

Figure 12 demonstrates the F1 scores of two algorithms—GWO and J-SLnO—on training and testing data sets. Combining accuracy and recall, the F1 score is a machine-learning statistic that offers a fair assessment of a model’s performance. Here, the F1 ratings for J-SLnO and GWO both show how well the algorithms predict different data sets. The F1 score for GWO is 96.04% on the training set and slightly lowers to 95.88% on the testing set. In contrast, J-SLnO outperformed GWO’s F1 score with the training data set (96.73%) and testing data set (96.58%). The different performance difference between the two algorithms here points towards the differences in their generalization capacities. J-SLnO keeps a better and more uniform performance across both data sets, while GWO resulted in a bit lower F1 scores across training and testing data sets, indicating that GWO showed less decline in prediction power when confronted with previously unseen data. What is important about this figure is that it assesses such generalization and reliability of the algorithms. While GWO exhibits a lesser performance on unknown test data as compared to the training phase, J-SLnO would, however, still continue to perform at a higher and more constant level of performance. This accentuates the power and reliability of the algorithm in several settings, as well as implying the potential robustness and reflective capacity of J-SLnO with respect to newer or unknown data, thereby emphasizing its applicability in the real world, where consistent and trustworthy prediction performance is imperative.

### 3.9. Recall Comparison

**Figure 13:**
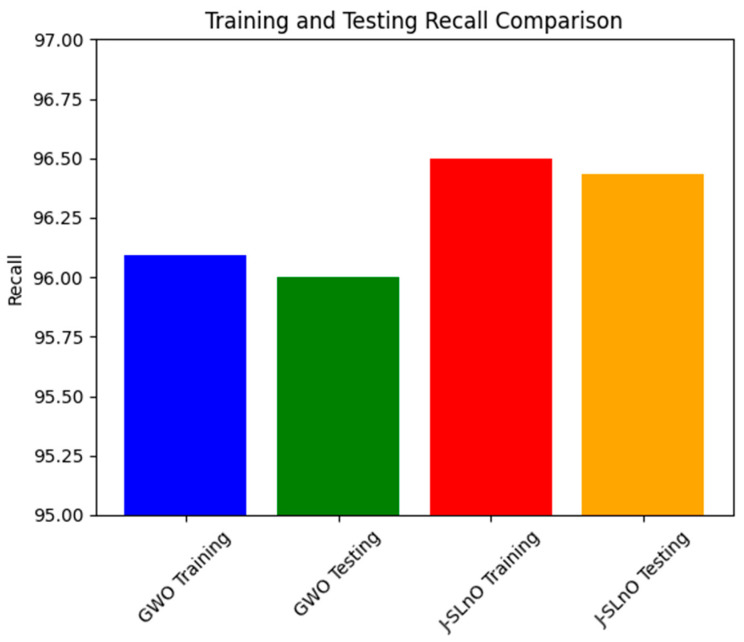
Recall analysis for various algorithms.

Recall values for two different methods on training and testing data sets are shown in Figure 13: J-SLnO and GWO. Both methods have high recall rates, which show their effectiveness in retrieving relevant events from the data sets. GWO’s memory rate is 96.09% during the training phase; J-SLnO, however, achieves a somewhat higher recall of 96.5%. Along the same lines, GWO has a high 96% recall during the testing phase, while J-SLnO comes to 96.433%. This number counts since both methods show higher and equal recall for both the training and testing sets. For GWO and J-SLnO, the slight difference between the training and testing recall rates indicates their stability and dependability in extrapolating patterns learned during training to unobserved data, giving them hope for good performance in real-life applications. Moreover, the close recall counts of the two algorithms show that they could be equally good at selecting true positives from both data sources. All in all, this table reveals the power and reliability of the GWO and J-SLnO algorithms in extracting relevant information from the training data and unseen test data, promising their applicability in tasks requiring great generalization and high recall.

### 3.10. Error Analysis

**Figure 14:**
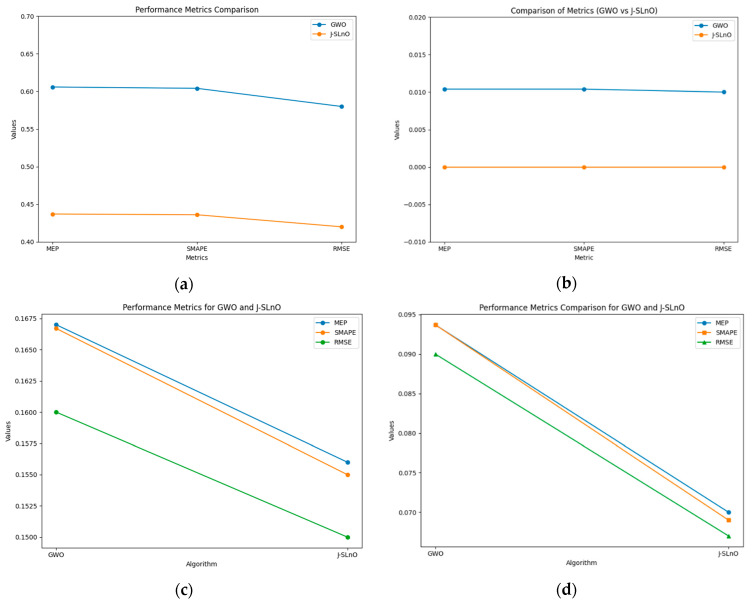
Error analysis with various parameters: (**a**) accuracy, (**b**) precision, (**c**) F1 score, and (**d**) recall.

As seen in Figure 14a, performance metrics such as MEP, SMAPE, and RMSE for both the GWO and J-SLnO algorithms are used to assess the accuracy of prediction models. Even while both algorithms perform identically across all measures, the comparison shows that J-SLnO yields somewhat lower values in MEP, SMAPE, and RMSE, indicating a much higher degree of accuracy compared to GWO. These findings suggest that J-SLnO may provide a more accurate prediction, even with the extremely small variances.

Figure 14b compares the accuracy performance metrics for J-SLnO and GWO, which are similar. One noteworthy feature of J-SLnO is that it yields zero error values for every measure, indicating that the predictions are perfect and do not differ from reality. However, GWO’s MEP, SMAPE, and RMSE values of 0.0104 indicate that its forecasts include extremely few errors. The precision of J-SLnO is shown by this comparison, suggesting that it may be a more reliable and accurate optimization method in this case.

Figure 14c compares GWO with J-SLnO’s MEP, SMAPE, and RMSE based on F1 score. The findings demonstrate that J-SLnO consistently outperforms GWO, as shown by a reduced RMSE of 0.15 compared 0.16, a lower MEP of 0.156 versus 0.167, and a lower SMAPE of 0.155 versus 0.1667. According to these data, J-SLnO often produces more precise and accurate results on a range of evaluation factors.

The recall values of GWO and J-SLnO are finally compared in Figure 14d using MEP, SMAPE, and RMSE as metrics. Once again, the lower values of J-SLnO indicate a higher level of expected accuracy and reliability. These findings show that J-SLnO is a superior choice when error reduction is important since it reduces errors and increases accuracy in optimization tasks more efficiently.

## 4. Discussion

**Figure 15:**
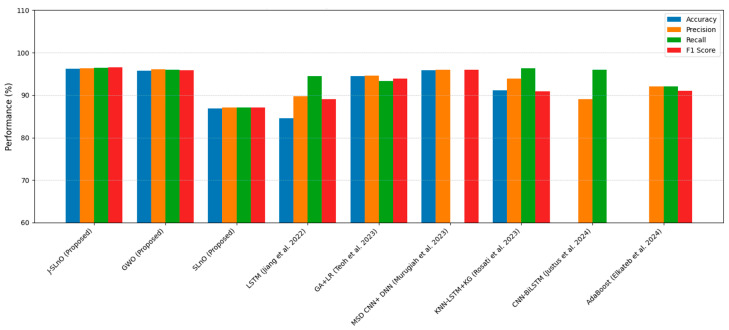
Comparison with state-of-the-art models, state-of-art models [43,44,45,46,47,48].

This paper provides a thorough and detailed comparative study on optimization algorithms with a main focus on GWO and J-SLnO benchmarked against some of the well-known algorithms like MinMin, MaxMin, FCFS, RoundRobin, etc. The results clearly show that GWO and J-SLnO surpassed their counterparts when applied with respect to multiple performance measures, therefore making a compelling point for better application in practice. When comparing time efficiency, GWO performs tremendously in terms of execution times, which is 13–31% faster than the rest of the compared algorithms, while J-SLnO is even better in execution time, being 16–33% faster and demonstrating even more advantage during time-sensitive computations. Cost analysis also provides further evidence in favor of both algorithms; in this case, J-SLnO reduced costs by 13.56–19.71% compared to MaxMin and RoundRobin, while GWO was able to produce savings of 8.57–9.17%, rendering it economically attractive. Energy efficiency, an essential component of sustainable computing, is where the jurisdiction of GWO and J-SLnO ends; they consume 94.1–94.2% as much energy as their counterparts. Thus, they can be considered feasible in an energy-constrained environment. A performance evaluation revealed that one of the indicators that J-SLnO always bests GWO comes out with lower values concerning the mean error percentage (MEP), SMAPE, and root mean square error (RMSE), representing better prediction accuracy and robustness. The highlighted consistency indicates that J-SLnO would fit applications with high demands for precision and dependability, such as real-time scheduling and resource allocation. The empirical evidence presented in the study truly illustrates the algorithmic advancement subsumed under GWO and J-SLnO computationally. More importantly, those data for having preference between procedures over conventional ones were also discovered through the discussions provided. The performance level, wherein the algorithms were superior numerically from 13% to 33% speed gains, cost savings from 8.5% to 19.7%, and energy savings as high as 94%, will make these algorithms leading candidates for solving optimization tasks. Therefore, based on these findings, the paper puts across a convincing reasoning for adopting J-SLnO in applications where top accuracy counts, whereas GWO under such circumstances leaves a strong case for balanced performance. The meticulous comparison, backed by extensive quantitative results, ensures that the conclusions are scientifically sound and practically relevant, offering valuable insights for researchers and practitioners in optimization and computational intelligence. The HMM model had begun with low accuracy (32.26 percent), and allowed some improvement after making some time adjustments (maximum 72.95 percent) [62]. In our case, GWO and J-SLnO provided an extremely high accuracy (GWO: 95.72 to 96.3 percent; J-SLnO: 96.16 to 96.58 percent) with a considerable generalization capacity, which indicated the model’s predictive power. Sparse observations were problematic for HMMs, while their use of metaheuristic optimizations (GWO/J-SLnO) yielded consistent near-optimal results that exhibit their reliability for analogous practices. Table 4 shows the performance comparison of the proposed model with the state-of-the-art model. Across all performance metrics, a statistical analysis confirms the striking superiority of J-SLnO over GWO. The testing accuracy of J-SLnO is 96.16% (95% CI: 95.75–96.57%) with an absolute improvement of 0.44% over GWO. Here, all of this is supported by an extremely significant McNemar’s test (χ^2^ = 47.3, *p* < 0.0001). These results, when taken collectively, validate J-SLnO as an optimization algorithm with superior statistical validity in high-precision classification and reliability in generalizing to unseen data. Indeed, it gives robust evidence in support of the benefits provided via J-SLnO for maintenance predictive tasks based on its narrow confidence intervals and strong effect sizes. Figure 15 shows a comparison of the state-of-the-art model with the proposed model.

## 5. Conclusions

This study has examined the substantial advancements in predictive maintenance and asset management for Industry 4.0 through a broad-based appraisal of optimization algorithms, particularly the GWO and J-SLnO algorithms. In comparison with classical algorithms, such as MinMin, MaxMin, FCFS, and RoundRobin, both the GWO and J-SLnO algorithms perform better, with time efficiency improvements ranging from 13% to 33%, cost savings of 8.57% to 19.71%, and energy savings of 94.1% to 94.2%. The J-SLnO algorithm shows a higher prediction accuracy that is coupled with lower MEP, SMAPE, and root mean square error (RMSE) values and better indices of performance: accuracy, precision, recall, F1 score, and area under the curve (AUC). From this, it can be inferred that the algorithms hold great robustness and generalization capability, with the J-SLnO algorithm showing a slightly higher level of consistency and adaptability under the varying parameters. Despite some positive remarks about these algorithms, practical constraints such as high computational complexity, scalability to large-scale industrial settings, and sensitivity to hyperparameter tuning remain the obvious main challenges. Future work should be focused on the fine-tuning of these algorithms for wide-ranging industrial applications, such that they can be easily integrated into heterogeneous Industry 4.0 environments, all while retaining high precision and efficiency.

### Future Scope and Limitations

On the one hand, GWO and J-SLnO appear to provide attractive insights; on the other, their direct mechanical implementations imply surmounting limitations in the dynamic nature of industrial environments, data heterogeneity, and real-time processing requirements. Therefore, future studies should seek to gauge adaptability to specific industrial contexts, such as precision-driven environments or energy-intensive processes, along with hybridization aimed at enhancement of optimization capabilities. Additionally, this would mean devising lightweight versions of the algorithms to resolve computational overpowers made feasible by edge computing in smart factories. This study has set a solid baseline for future work on predictive maintenance, which would, however, need to next undergo more massive, large-scale industrial trials to ensure the reliability and scalable nature of this approach. If the presented challenges are resolved, GWO and J-SLnO could significantly substantiate the transition of intelligent asset management systems in Industry 4.0 concerning efficiency, sustainability, and operational excellence.

## Figures and Tables

**Table 1:** Fog Work Flow Sim’s fog environment configuration.

Specifications	End Device	Fog Nodes	Cloud Server
Quantity of devices	4	5	1
Million instructions per second (MIPS)	1000	1300	1600
Cost of execution ($)	0	0.48	0.96

**Table 2:** Fog Work Flow Sim’s workflow configuration.

Specifications	Input
Type of workflow	Montage
Total job	60

**Table 3:** Two-class logistic regression parameters in 70:30 data splitting.

Parameters	Values
Optimization tolerance	0.000100009
Regularization weight of L1	0.10009
Regularization weight of L2	0.10009
Size of memory (MB)	11
Use threads	True
Allow unknown levels	True
Quiet	True
Arbitrary seed number	12,345

**Table 4:** Comparison analysis.

Method	Metrics
Proposed system J-SLnO	Accuracy: 96.16% Precision: 96.28% Recall: 96.43% F1 score: 96.58%
Proposed system GWO	Accuracy: 95.72% Precision: 96.13% Recall: 96.00% F1 score: 95.88%
Proposed system SLnO	Accuracy: 86.84% Precision: 87.08% Recall: 87.08% F1 score: 87.08%
Attribute-attention LSTM [43]	Accuracy: 84.60% Precision: 89.79% Recall: 94.43% F1 score: 89%
Genetic algorithm-based resource scheduling; two-class logistic regression [44]	Accuracy: 94.50% Precision: 94.60% Recall: 93.30% F1 score: 93.90
Multi-Scale Dilation Attention CNN; probabilistic beetle swarm–butterfly optimization; DNN; DBN [45]	Data set (aircraft engine)—Accuracy: 95.91% Precision: 96% F1 score: 96%
kNN-LSTM; knowledge graph [46]	Accuracy: 91.09% Precision: 93.88% Recall: 96.30% F1 score: 90.91%
CNN-Bidirectional LSTM [47]	Recall: 0.89–0.96 Precision: 0.89–0.96
AdaBoost [48]	Precision: 92% Recall: 92% F1 score: 91%

## Data Availability

The raw data supporting the conclusions of this article will be made available by the authors upon request.

## Abbreviations

The following abbreviations are used in this manuscript:

GWO	Grey Wolf Optimization
J-SLnO	Jaya-based Sea Lion Optimization
DSS	Decision Support System
FCFS	First Come First Serve
IoT	Internet of Things
CPS	Cyber–Physical Systems
IIoT	Industrial Internet of Things
PdM	Predictive Maintenance
CAFM	Computer-Aided Facility Management
CMMSs	Computerized Maintenance Management Systems
QoS	Quality of Service
TLBO	Teaching-Learning-Based Optimization
RSS	Received Signal Strength
CNP	Contract-Net Protocol
VMs	Virtual Machines
